# Increase in human West Nile and Usutu virus infections, Austria, 2018

**DOI:** 10.2807/1560-7917.ES.2018.23.43.1800545

**Published:** 2018-10-25

**Authors:** Stephan W. Aberle, Jolanta Kolodziejek, Christof Jungbauer, Karin Stiasny, Judith H. Aberle, Alexander Zoufaly, Michael Kai Hourfar, Lisa Weidner, Norbert Nowotny

**Affiliations:** 1 Center for Virology, Medical University of Vienna, Vienna, Austria; 2 These authors contributed equally to this article and share first authorship; 3 Viral Zoonoses, Emerging and Vector-Borne Infections Group, Institute of Virology, University of Veterinary Medicine Vienna, Vienna, Austria; 4 Austrian Red Cross, Blood Service for Vienna, Lower Austria and Burgenland, Vienna, Austria; 5 Department of Medicine IV, Kaiser Franz Josef Hospital, Vienna, Austria; 6 German Red Cross, Blood Donor Service Baden-Württemberg-Hessen, Institute for Transfusion Medicine and Immunohaematology, Johann Wolfgang Goethe University, Frankfurt am Main, Germany; 7 Department of Basic Medical Sciences, College of Medicine, Mohammed Bin Rashid University of Medicine and Health Sciences, Dubai, United Arab Emirates

**Keywords:** West Nile virus, West Nile fever, West Nile neuroinvasive disease, Usutu virus, blood donor, Austria

## Abstract

Between 28 June and 17 September 2018, 27 cases of human West Nile virus infections were recorded in Austria; four cases of West Nile neuroinvasive disease, 11 cases of West Nile fever, six infections detected by blood donation screening and six imported cases. In addition, 18 cases of human Usutu virus infections (all blood donors) were recorded. This is the highest number of annual infections recorded in Austria since the introduction of both viruses.

West Nile virus (WNV) and Usutu virus (USUV) are closely related mosquito-borne viruses (genus *Flavivirus*; family *Flaviviridae*). WNV infection in humans may result in disease of varying severity, from West Nile fever (WNF) to possibly lethal West Nile neuroinvasive disease (WNND) [1]. Human infections with USUV are usually asymptomatic or occasionally associated with rash [2]; severe disease is rarely seen and occurs mainly in immunocompromised patients [3,4]. In 2018, the highest ever number of WNV and USUV infections were detected in Austria.

## Human West Nile virus and Usutu virus infections in Austria, 2009–2018

The first three human West Nile disease (WND) cases were identified in eastern Austria in 2009 and 2010; two cases of WNND and one case of WNF [5]. Since then, autochthonous human WND cases have been diagnosed every year, with the exception of 2011–2013, when only imported WNV cases were detected (Table 1).

**Table 1:** Number of diagnosed human West Nile and Usutu virus infections, Austria, 2009–2018

Year	West Nile virus infections						Reference	Usutu virus infections	Reference
	Autochthonous				Imported	Total		Autochthonous	
	WNND	WNF	Asympt WNV	Blood donors				Blood donors	
2009	1	1	0	ND	0	2	[5]	ND	NA
2010	1	0	0	ND	0	1		ND	NA
2011	0	0	0	ND	0	0	NA	ND	NA
2012	0	0	0	ND	3	3	NA	ND	NA
2013	0	0	0	ND	0	0	NA	ND	NA
2014	1	0	0	1	0	2	[7,8]	0	NA
2015	1	0	1	5	0	7	[6] [9]	0	NA
2016	1	1	0	3	0	5		1	[9]
2017	2	2	1	1	1	7		6	
2018	4	11	0	6	6	27	NA	18	NA
**Total**	**11**	**15**	**2**	**16**	**10**	**54**	**NA**	**25**	**NA**

Asympt: asymptomatic; NA: not applicable; ND: not done; WNF: West Nile fever; WNND: West Nile neuroinvasive disease; WNV: Nest Nile virus.

**Figure 1:**
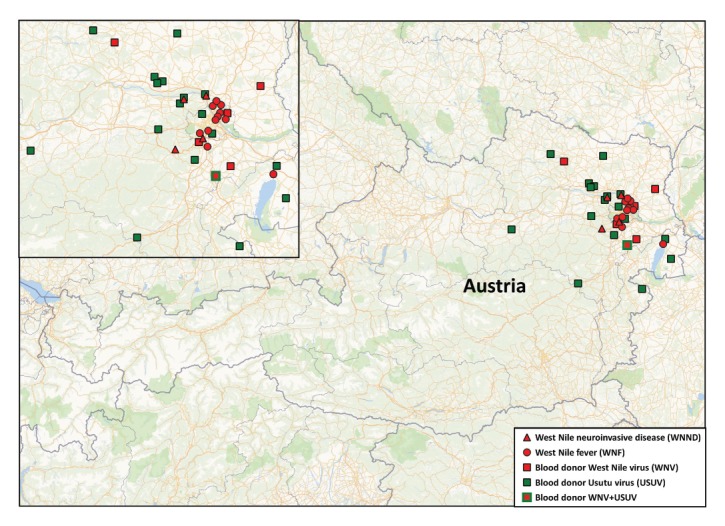
Geographic distribution of human West Nile and Usutu virus infections, Austria, 2018

Between 28 June and 17 September 2018, 27 cases of human WNV infections were recorded of which 21 were locally acquired. Four cases were WNND, 11 cases were WNF, six were WNV infections detected by blood donation screening and six cases were associated with travels to Serbia (n = 2), Italy (n = 1), Greece (n = 1), Hungary (n = 1), and Croatia (n = 1). USUV infections were identified in 18 of 31,598 blood donations tested (Table 1). All autochthonous WNV and USUV infections were acquired in eastern Austria (Figure 1).

In Austria, human, veterinary and entomological surveillances to detect WNV and USUV include seasonal testing of blood donations originating from endemic areas, detailed sample examination of humans and equids with neurological symptoms, passive ornithological monitoring as well as regular, nationwide mosquito surveillance (for details see [6]).

In 2014, the Blood Service for Vienna, Lower Austria and Burgenland of the Austrian Red Cross introduced an automated nucleic acid test (NAT) on the cobas 8800 system (cobas WNV assay; Roche, Rotkreuz, Switzerland) for all blood donated between 1 June and 30 November each year. Already in the first year of screening, one WNV-positive donation was identified [7,8] as well as one clinical WNND case (Table 1). In the 2015/16 [6] and 2017 seasons, further clinical WNV cases were diagnosed and positive blood donations were identified [9]. To date, no WND associated fatalities have occurred in Austria. Increasing USUV activity in birds in recent years in Austria was reflected in human infections, when one of four, and six of seven flavivirus NAT-positive blood donations turned out to be USUV positive and not WNV positive in 2016 and 2017, respectively (Table 1) [9].

In 2018, overall, 15 patients and three blood donors showed signs and symptoms compatible with WNV infection (Table 2). Three of four patients with WNND were male and the median age was 62 years (range 58–77). Of the 11 WNF cases, six were male and the median age was 51 years (range 33–87). The overall median age of the blood donors was 56 years (range 23–71) with no difference between WNV and USUV-positive persons. Five of six and 14 of 18 WNV and USUV-positive blood donors, respectively, were male. Of the 18 USUV infections identified from blood donations tested, 16 donors remained asymptomatic, one developed a rash and one donor did not disclose information. One blood donor had a dual infection with WNV and USUV (Table 2).

**Table 2:** Details of autochthonous human West Nile and Usutu virus infections, Austria, 2018

Cases	Main symptoms	Month of symptom onset/ blood donation	Cobas WNV assay^a^	WNV RT-PCR^b^	USUV RT-PCR^b^	WNV NT	USUV NT
**WNND**							
1	Disorientation, aphasia	August	ND	Pos	Neg	240	40
2	Disorientation, coma	September	ND	Pos	Neg	640	60
3	Fever, stiff neck	August	ND	Pos	Neg	160	20
4	Dysarthria, limb weakness	September	ND	Neg	Neg	240	60
**WNF**							
1	Fever, rash, fatigue, headache	August	ND	Pos	Neg	960	160
2	Fever, rash, diarrhoea	August	ND	Pos	ND	320	80
3	Fever, rash	August	ND	Pos	Neg	640	60
4	Rash	August	ND	Pos	ND	ND	ND
5	Fever, rash	August	ND	Pos	ND	40	30
6	Fever, rash	August	ND	Pos	ND	320	120
7	Rash, ocular pain	August	ND	Pos	ND	320	40
8	Fever, fatigue, muscle aches	August	ND	Pos	ND	480	60
9	Rash, fatigue	August	ND	Neg	Neg	240	60
10	Fever, rash	August	ND	Pos	ND	120	< 20
11	Fever, fatigue	August	ND	Pos	Neg	320	< 20
**Blood donors**							
**WNV**							
BD2	Rash, fatigue, headache^c^	August	Pos (30.4)	Pos	Neg	480	30
BD4	Rash, fatigue^d^	August	Pos (40.0)	Pos	Neg	1,920	160
BD9	Fatigue, joint aches^c^	August	Pos (30.8)	Pos	Neg	240	80
BD17	Asympt	August	Pos (30.0)	Pos	Neg	160	60
BD18	Asympt	August	Pos (39.4)	Pos	Neg	240	160
**WNV/USUV**							
BD7	Asympt	August	Pos (34.1)	Pos	Pos	960	120
**USUV**							
BD1	Asympt	June	Pos (38.7)	Neg	Pos	< 20	40
BD3	Rash^c^	August	Pos (37.6)	Neg	Pos	60	160
BD5	Asympt	August	Pos (39.4)	Neg	Pos	ND	ND
BD6	Asympt	August	Pos (37.1)	Neg	Pos	80	240
BD8	Asympt	August	Pos (44.0)	Neg	Pos	< 20	80
BD10	Asympt	August	Pos (35.3)	Neg	Pos	ND	ND
BD11	Asympt	August	Pos (38.8)	Neg	Pos	30	120
BD12	Asympt	August	Pos (40.7)	Neg	Pos	< 20	160
BD13	Asympt	August	Pos (38.4)	Neg	Pos	< 20	40
BD14	No information	August	Pos (39.3)	Neg	Pos	ND	ND
BD15	Asympt	August	Pos (36.5)	Neg	Pos	ND	ND
BD16	Asympt	August	Pos (36.8)	Neg	Pos	20	40
BD19	Asympt	August	Pos (36.6)	Neg	Pos	20	120
BD20	Asympt	August	Pos (42.1)	Neg	Neg	< 20	80
BD21	Asympt	August	Pos (41.7)	Neg	Neg	< 20	80
BD22	Asympt	September	Pos (40.0)	Neg	Pos	ND	ND
BD23	Asympt	Septenber	Pos (38.6)	Neg	Pos	40	240

Asympt: asymptomatic; ND: not done; Neg: negative; NT: Neutralizing antibody titer in first or follow-up serum sample; Pos: positive; USUV: Usutu virus; WNF: West Nile fever; WNND: West Nile neuroinvasive disease; WNV: West Nile virus.

^a^Nucleic acid test performed in plasma samples. Cq values obtained with pools of 19 donations are shown in brackets.

^b^WNV and USUV PCR performed in plasma, whole blood, serum, urine and/or cerebral spinal fluid.

^c^Symptoms occurred 1–3 days after blood donation. The donors were viremic but asymptomatic.

^d^Mild symptoms 2 days before blood donation, but asymptomatic at blood donation.

All infections diagnosed up to 24 October 2018 are included.

WNV and USUV PCRs, as well as neutralisation tests, were performed as previously described [5,6,9].

## Sequencing information

**Figure 2:**
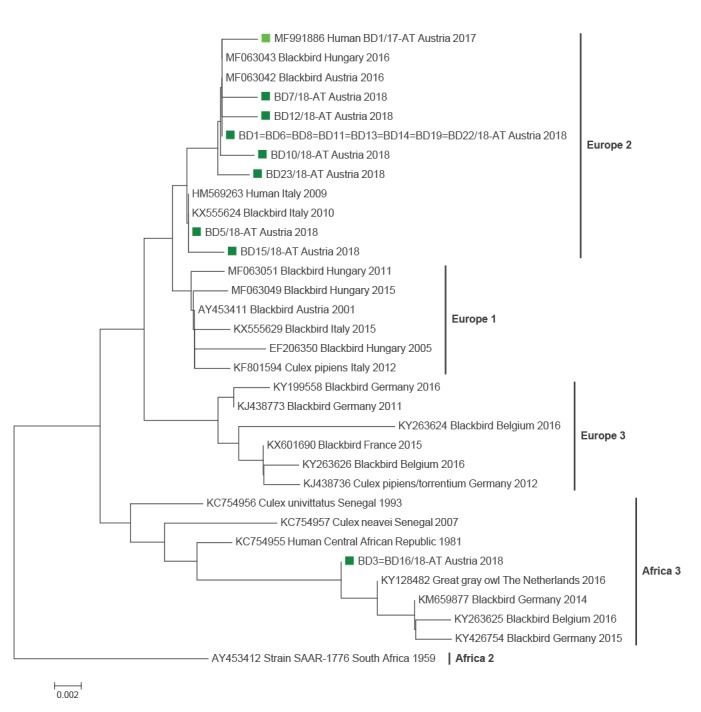
Phylogram demonstrating the genetic relationships among Usutu viruses based on partial (396 nts) NS5 protein coding nucleotide sequences

Sequencing of short fragments of the WNV C/prM (294nts) and USUV NS5 (396nts) genes enabled exact identification of the detected viruses. This analysis revealed that all WNV strains belonged to lineage 2 with a 98.98–100% identity to the Austrian strain BD2/2016 [6] (GenBank accession number MF984347). All but two USUV strains belonged to the currently in Austria most widespread ‘Europe 2’ lineage showing 99.84–100% identity to European 2 sequences of Hungarian blackbirds (e.g. GenBank accession number MF063048) and an Austrian blood donor sequence from 2017 (GenBank accession number MF991886)(Figure 2). The two USUV strains from BD3 and BD16, however, clustered within the ‘Africa 3’ lineage and were 99.52-99.84% identical to the African 3 sequences of birds from Germany and The Netherlands (e.g. GenBank accession numbers KY294723, KY128482)(Figure 2).

BD: blood donor.

The phylogenetic tree was created by the neighbour-joining method with the MEGA7 program as described previously [6]. Usutu virus sequences determined in this study are marked with dark green squares and one sequence from 2017 [9] with a light green square. Sequences of eight samples in cluster Europe 2 and sequences of two samples in cluster Africa 3 are genetically identical to each other and cluster therefore together, respectively. GenBank accession numbers of USUV sequences derived from 16 blood donors were submitted on 22 October 2018 and will be added when available.

## Discussion

With 21 locally-acquired and six imported human WNV infections the 2018 transmission season has been the most relevant since the introduction of the aetiological lineage 2 WNV to Austria in 2008 [10,11]. The number of autochthonous human infections identified so far in 2018 has almost reached the cumulative number diagnosed in the past 10 years (2008–2017) in eastern Austria (23 locally-acquired human infections). In addition, obligatory seasonal blood donation screening and subsequent testing by virus-specific RT-PCR assays revealed 18 USUV infections among blood donors, which is the highest number of human infections reported since the emergence of USUV in Austria in 2001 [12]. An early start of the WNV/USUV transmission season was observed in Austria in 2018, which (at least for WNV) was also seen in other European countries [13,14] and might have been associated with favourable environmental and climatic conditions for an early upsurge of the vector population [14].

One blood donor was found to have a double infection with both WNV and USUV for the first time. While the central European lineage 2 [15] was the causative WNV strain in all sequenced cases, two different USUV lineages were identified in the blood donors. The vast majority were infected with USUV lineage ‘Europe 2’, which is currently the dominating USUV strain in Austria [9,16]. Two donors, however, were infected with the USUV ‘Africa 3’ lineage, which is also circulating in Austria on a smaller scale (unpublished data). Follow-up investigations of WNV positive blood donors revealed mild symptoms e.g. rash and fatigue in three of the six cases a few days before or after donating blood, whereas 16 of 18 USUV-RNA positive donors did not report any symptoms; one donor reported a rash and for one case no information was available. Although USUV seems to be less pathogenic for humans than WNV, the virus might cause severe disease in immunocompromised patients [3,4] or might be involved in other neurologic disorders such as idiopathic facial paralysis, as recently reported from France [17].

USUV is spreading in Europe, which may lead to increased numbers of human infections. In countries with blood donation testing for flavivirus RNA, health authorities should be aware that positive WNV screening results could be due to USUV infections and have to be further differentiated. In addition, countries where blood donation testing is not performed for flaviviruses, USUV might be transmitted through contaminated blood units. Although no transfusion-associated USUV infection has been reported so far, it is of utmost importance to further investigate the clinical relevance of USUV infections.
